# The impact of sensory modalities and background information on the emotional resonance of Li Bai’s classical poetry

**DOI:** 10.3389/fpsyg.2025.1541680

**Published:** 2025-07-01

**Authors:** Yannan Zhang, Xiao Wang

**Affiliations:** College of Arts, Northeastern University, Shenyang, China

**Keywords:** sensory modalities, background information, poetry, emotional resonance, Li Bai

## Abstract

**Introduction:**

Emotional resonance (ER) refers to the extent to which readers identify with and emotionally connect to the representations in poetry, often drawing parallels between the emotions conveyed in the poem and their own personal experiences. This phenomenon is influenced by sensory modalities—such as visual, auditory, tactile, gustatory, and olfactory—and the cultural context embedded within the poem. Understanding how these factors interact to enhance emotional resonance in poetry is crucial for advancing poetry education and engagement.

**Methods:**

This study employs a repeated-measures experimental design to investigate how different sensory modalities and contextual background influence emotional responses to classical poetry. The experiment includes five conditions: three focus on sensory modalities (visual, auditory, and visual-auditory), while the remaining two examine the combined effects of background information (background music and background images) in conjunction with sensory modalities.

**Results:**

The results indicate that: The visual modality and the visual-auditory modality yield higher emotional resonance ratings compared to the auditory modality. Background information (background music and images) enhances the emotional resonance of the poetry. The synergy between background information and sensory modalities further increases the emotional resonance ratings.

**Discussion:**

This study highlights the critical role of both sensory modalities and background information in shaping the emotional response to poetry. The findings suggest that integrating different sensory experiences and cultural cues can significantly enhance emotional resonance, offering new theoretical insights for the teaching and appreciation of poetry.

## Introduction

Blizek (1973) proposed the aesthetic motivation theory, which argues that emotional resonance (ER) in art stems from its ability to evoke emotions in the viewer or reader. In poetry, imagery represents the manifestation of abstract emotions created within subjective consciousness, symbolizing objects that carry emotional weight (Frame et al., 2024). The stronger the ER elicited by an artwork, the deeper the reader’s aesthetic experience of its imagery. Other studies have also suggested that ER enhances the reader’s aesthetic evaluation (Blizek, 1973), and recent research indicates that ER significantly contributes to the aesthetic appeal of art (Silvia, 2005). Santos et al. (2021) further pointed out that ER induced by art enhances aesthetic pleasure.

Specifically, in the field of poetry, poetry attracts readers’ attention through its unique aesthetic qualities, initially sparking their interest and curiosity about the appreciation of beauty. Through profound emotional expression and the creation of artistic imagery, poetry gradually resonates with the reader’s inner world, eliciting emotional engagement and reflective thinking (Leder et al., 2014). ER refers to the reader’s ability to find similarities between their own emotions or experiences and those conveyed in poetry, thereby fostering a deep emotional connection and sense of identification (Graber and Sumera, 2020; Liu X. L., 2022). It can be understood as the extent to which an individual identifies with and comprehends the emotional representations in poetry (Coburn, 2001). ER is influenced by an individual’s relevance to the emotional aspects of poetry, including the modalities through which they receive and process external information. These modalities primarily include visual, tactile, auditory, gustatory, and olfactory modalities (Frame et al., 2024). Additionally, ER is shaped by the cultural background embedded in poetry (Yuan and Guoyuan, 2022).

### The impact of sensory modalities on the emotional resonance of poetry

ER typically occurs within milliseconds and is determined by the “interactive structure,” which influences the extent to which a relatively definite feeling or a sense of mutual authenticity is experienced (Stolorow and Atwood, 2014). Previous research has primarily examined the role of ER in the visual modality (Beebe et al., 1997; Coburn, 2001; Pan et al., 2019).

Specifically in the field of poetry, previous research comparing the effects of different sensory modalities on ER has been limited. Ongoing self-reports on the impact of spoken poetry on aesthetic evaluation and ER showed no significant differences in ratings and the distinction between spoken and written poetry has yet to be thoroughly examined (Wagner et al., 2021). Many studies have shown that, compared with pure audio or pure visual presentation, the combination of audio and visual information can enhance emotional perception and appeal (Thompson et al., 2008). A recent study demonstrated that sensory modalities, as important channels of information perception, produce differences in the intensity of ER across modalities (Bishop, 2019). Overall, the finding regarding the limited comparisons among visual, auditory, and audiovisual modalities remain unclear (Frame et al., 2024).

### The impact of background information on the emotional resonance of poetry

The concept of culture is an abstract notion that evokes differences by separating subjectivity, identity, and language from lived behaviors (O’Connor, 2024). Artists engage with culture, and culture, in turn, influences individuals, generating ER (Paiva, 2020).

Specifically in the field of poetry, research has primarily focused on poetry education. Previous research found that the signifiers of background music and its direct identity evoke an immediate intuitive understanding (Gu, 2024), and music in poetry often evoked stronger ER (Küssner and Eerola, 2019). Similarly, the symbols, composition, and colors of background images can convey specific cultural emotional values and modes of expression (Langacker, 2002). In the process of poetry education, background images enhance the ER of poetry (Zhao, 2024). However, research using experimental methods to compare the impact of different cultural backgrounds on ER remains limited (Wang and Wang, 2024).

### Aims

Based on the above discussion, ER is the phenomenon in which individuals perceive the emotions of others, causing a change in their own emotional state. Sensory modalities serve as important channels through which humans perceive external information (Frame et al., 2024). Previous research has shown that emotional intensity varies depending on the modality of information reception (Johnson-Laird and Oatley, 2022). This study focuses on a limited set of sensory modalities (visual, auditory, and visual-auditory) and compares their impact on ER in poetry.

Moreover, existing studies have found that ER is greatly influenced by cultural background (Alshaer, 2025). Since individual development is significantly affected by social norms and cultural traditions (Guo, 2024), people tend to show stronger ER when exposed to situations belonging to their own cultural background (Miller, 2015).

Therefore, the hypotheses of this study are as follows:

H1: Sensory modalities (visual, auditory, and visual-auditory) differ in their impact on the ER of poetry.

H2: Culture background information (background music and background image) enhances the ER of poetry.

H3: Culture background information synergizes with sensory modalities, collectively enhancing the ER of poetry.

These hypotheses form the core framework of this study, which aims to explore how different dimensions of sensory modalities and background information individually and synergistically affect the ER of poetry, offering new perspectives for research in related fields.

## Materials and methods

### Participants

In our study, thirty-seven participants completed the experiment, including five males (22.00 ± 3.94 years) and thirty-two females (18.40 ± 1.81 years). Using G*Power calculations, a sample size of thirty-seven yielded a statistical power of 0.96 under an effect size of f = 0.25 and a level of α = 0.05, demonstrating that the study was both practical and adequately powered.

All participants were right-handed, had normal or corrected vision without any visual impairments or color blindness and had none reported any history of neurological conditions or nerve-related disorders. The participants were all students from the School of Fine Arts, with Mandarin Chinese as their native language. They had received training in the poetry of Li Bai during their primary and secondary education in China, which enabled them to have a profound understanding of the rhythm, rhyme, and connotations of Li Bai’s poetry (Li et al., 2009). Each participant was compensated with 10 yuan for their involvement after the experiment.

### Ethics approval and consent to participate

This study was approved by the Institutional Review Board (IRB) of Northeastern University, China. Prior to participation, all participants provided written informed consent, confirming their voluntary involvement and full comprehension of their rights. All research procedures strictly adhered to the ethical standards outlined in the Belmont Report and the Declaration of Helsinki, in compliance with relevant ethical guidelines and regulations.

### Stimuli and procedure

**FIGURE 1:**
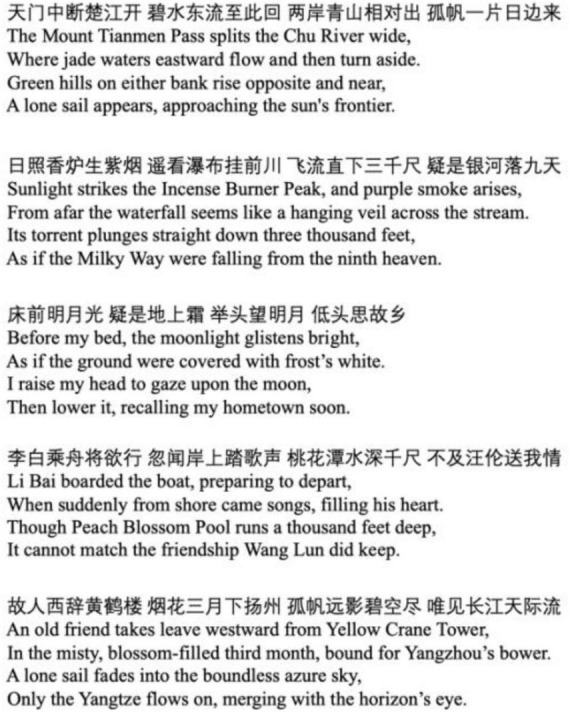
Sample of poetry.

As shown in Figure 1, the study used a total of five poems by Li Bai. The poems were selected based on three criteria: first, relative consistency in emotional tone, choosing works with calm and serene tone to match the neutral reading style; second, thematic diversity, covering common emotional and philosophical themes in Li Bai’s poetry, such as admiration of nature, homesickness, and friendship; third, poem length ranging between 20 and 30 Chinese characters, which ensured moderate length and comparable cognitive load across poems. Every poem was delivered by the same female voice, generated by AI with a neutral tone and no emotional inflections, including: Tianmen Mountain (天门山), Gazing upon the Waterfall at Mount Lu (望庐山瀑布), Reflections during a Tranquil Night (静夜思), A Farewell to Wang Lun (赠汪伦), and Bidding Meng Haoran Goodbye at Yellow Crane Tower (黄鹤楼送孟浩然之广陵). The audio files had an average duration of 7.15 s (SD = 0.83 s), an average sound pressure level (SPL) of 27.10 dB(A), and a speech rate of approximately 3.68 syllables per second.

**FIGURE 2:**
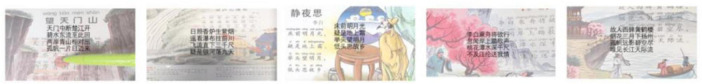
Sample of background images.

As shown in Figure 2, five background images related to Li Bai’s poems were utilized. The selected poems express Li Bai’s deep love for mountains and rivers, his admiration for nature, his longing for his hometown, his profound feelings of friendship, and his reluctance to part with friends. Participants evaluated the extent to which they perceived the emotional dispositions of the poetry and achieved ER with the poet, using a seven-point scale ranging from complete disagreement to complete agreement. Finally, the background music consisted of traditional Chinese guqin music, including: The Resonance of the Qin and Soul (琴心共鸣), Hearing Rain Amidst the Hills (半山闻雨), Song of Qingwen (晴雯吟), The Orchid-Scented Ravine (兰香涧), and Seated Contemplation of the Misty Peaks (坐看云峰). All music was purely instrumental with a neutral emotional tone, avoiding interference with participants’ emotions. The music had an average tempo of 101 beats per minute (BPM), and playback volume was controlled at 28.52 dB to maintain an acoustic environment consistent with the poetry recordings.

**FIGURE 3:**
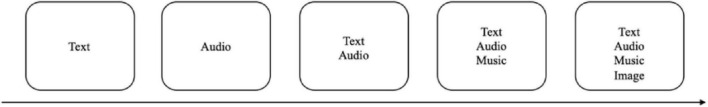
Five conditions of experimental procedure.

As shown in Figure 3, the experiment comprised two parts: the pilot study and the formal experiment. In the pilot study, to minimize the impact of existing knowledge and help soothe the participants’ emotions, they were asked to familiarize themselves with the experiment in the same setting as the formal experiment. Subsequently, participants were asked to rate their familiarity with the textual poetry on a seven-point scale. In the formal experiment, participants completed the experiment based on five conditions.

Condition 1 (visual): with the five poetic texts in Figure 1, each trial randomly presented one on the computer screen.

Condition 2 (auditory): with the audio versions of the five poetic texts, each trial randomly presented one on the computer screen.

Condition 3 (visual-auditory): With five poetic texts in Figure 1 and the corresponding audio versions, each trial randomly presented one on the computer screen.

Condition 4 (visual-auditory and background music): With five poetic texts in Figure 1 and the corresponding audio versions, each trial randomly presented one on the computer screen. Additionally, a randomly selected piece of background music from guqin music.

Condition 5 (visual-auditory, background music, and background image): With five poetic texts in Figure 1 and the corresponding audio versions, and the corresponding background image in Figure 2, each trial randomly presented one on the computer screen. Additionally, a randomly selected piece of background music from guqin music.

At the end of each trial, using a 7-point Likert scale, participants evaluated the poem’s ER. After submitting their response, they proceeded to the next trial. The experiment was carried out using Microsoft PowerPoint and automatically recorded via a linked Excel spreadsheet. All experimental were completed within a continuous time on the same day. Depending on individual pacing, the entire experiment took approximately 10 to 20 min. Throughout the experiment, silence was maintained. If participants had any questions, the experimenter focused on providing reassurance and encouraged participants to rate their feelings based on their genuine experiences. After the experiment concluded, five participants were randomly selected to inquire about their subjective feelings. Through verbal questioning, the experimenter recorded their main points in writing.

## Results with poetic texts

### Familiarity with poetic texts

On the familiarity evaluation of the textual poems, the participants’ average rating was 33.95 (SD = 2.32). This indicated that the participants had a high level of familiarity with the five poetic texts.

### Correlation analysis

As shown in Table 1, the ER ratings exhibited strong positive correlations across the five conditions. Condition 1 and Condition 2 showed a significant correlation (*r* = 0.600, *p* < 0.01), as did Condition 1 and Condition 3 (*r* = 0.593, *p* < 0.01), Condition 1 and Condition 4 (*r* = 0.679, *p* < 0.01), and Condition 1 and Condition 5 (*r* = 0.673, *p* < 0.01). Condition 2 and Condition 3 were also significantly correlated (*r* = 0.882, *p* < 0.01), as were Condition 2 and Condition 4 (*r* = 0.602, *p* < 0.01) and Condition 2 and Condition 5 (*r* = 0.682, *p* < 0.01). Similarly, Condition 3 and Condition 4 showed a significant correlation (*r* = 0.701, *p* < 0.01), as did Condition 3 and Condition 5 (*r* = 0.779, *p* < 0.01). Finally, Condition 4 and Condition 5 were significantly correlated (*r* = 0.759, *p* < 0.01). Overall, these results indicate that the emotional resonance of the poetry remained largely stable across different sensory modalities and background information conditions.

**TABLE 1:** Correlational analysis.

Condition	1	2	3	4	5
1					
2	0.600				
3	0.593	0.882			
4	0.679	0.602	0.701		
5	0.673	0.682	0.779	0.759	

### Analysis of variance

As shown in Table 2, a one-way repeated measures ANOVA indicated a significant effect across the five conditions [F (4, 144) = 16.821, *p* < 0.001, η^2^ = 0.318] and *post hoc* comparisons were conducted. The ratings of ER were higher when participants were presented with Condition 1 than with Condition 2 (MD = 2.162, *p* = 0.004). The ratings were higher when participants were presented with Condition 3 than with Condition 2 (MD = 1.784, *p* < 0.001). No significant difference was found between Condition 1 and Condition 3 (MD = 0.378, *p* = 0.563). Overall, in line with hypothesis H1, audiovisual and visual modalities resulted in higher ratings for ER compared to the auditory modality.

**TABLE 2:** Paired comparisons.

Condition		MD	SE	*P*	CI	
					Lower	Upper
1	2	2.162	0.712	0.004	0.718	3.606
	3	0.378	0.648	0.563	−0.935	1.692
	4	−2.054	0.478	< 0.001	−3.023	−1.085
	5	−1.622	0.547	0.005	−2.731	−0.512
2	1	−2.162	0.712	0.004	−3.606	−0.718
	3	−1.784	0.419	< 0.001	−2.634	−0.933
	4	−4.216	0.714	< 0.001	−5.663	−2.769
	5	−3.784	0.661	< 0.001	−5.125	−2.443
3	1	−0.378	0.648	0.563	−1.692	0.935
	2	1.784	0.419	< 0.001	0.933	2.634
	4	−2.432	0.569	< 0.001	−3.586	−1.279
	5	−2.000	0.511	< 0.001	−3.037	−0.963
4	1	2.054	0.478	< 0.001	1.085	3.023
	2	4.216	0.714	< 0.001	2.769	5.663
	3	2.432	0.569	< 0.001	1.279	3.586
	5	0.432	0.480	0.374	−0.541	1.406
5	1	1.622	0.547	0.005	0.512	2.731
	2	3.784	0.661	< 0.001	2.443	5.125
	3	2.000	0.511	< 0.001	0.963	3.037
	4	−0.432	0.480	0.374	−1.406	0.541

The ratings were higher when participants were presented with Condition 4 than with Condition 3 (MD = 2.432, *p* < 0.001), and also higher than Condition 1 (MD = 2.054, *p* < 0.001). Ratings were higher when participants were presented with Condition 5 than with Condition 3 (MD = 2.000, *p* < 0.001) and Condition 1 (MD = 1.622, *p* = 0.005). No significant difference was found between Condition 4 and Condition 5 (MD = 0.432, *p* = 0.374). Overall, consistent with hypotheses H2 and H3, background information beyond sensory modalities enhanced ratings of ER for the poetry (Supplementary Table 1).

### Qualitative analysis

In line with hypotheses H1, H2, and H3, participants generally expressed that the textual poetry allowed them to more directly perceive the aesthetic features of the poetry, making it easier to evoke ER. In contrast, auditory forms of poetry, due to the transient nature of auditory information and the potential for attention to be diverted, made it difficult for participants to capture specific poetic content, with ER occurring more slowly. Furthermore, background music helped engage participants’ emotions, while background images enhanced visual perception and increased the sense of immersion. This made the poetic imagery more vivid and intuitive, leading to stronger ER with the poetry.

## Discussion

### The impact of sensory modalities on the emotional resonance of poetry

In line with hypothesis H1, our study found that the audiovisual modality and the visual modality had a greater impact on ER in poetry compared to the auditory modality.

#### Visual modality

The first possible explanation is the objectivity of visual information, which can present the scenes and details in the poetry, allowing readers to more deeply feel the emotions conveyed in the text (Mehl et al., 2023). For example, in the sentence “天门中断楚江开, 碧水东流至此回” (The Tianmen Pass splits the Chu River wide, Where jade waters eastward flow and then turn aside.), the word “断” (splits) directly separates the river, which helps readers immediately grasp the emotional disruption and fluctuation.

The second possible explanation is the richness of visual cues, which can stimulate readers’ imagination and emotions (Jelinčić and Šveb, 2021). For instance, in the sentence “两岸青山相对出, 孤帆一片日边来” (Green hills on either bank rise opposite and near, A lone sail appears, approaching the sun’s frontier.), the imagery of green hills, lone sail, setting sun, and both banks provides rich visual elements, helping readers gain a deeper understanding of ER.

The third explanation may be that, during the process of reading a poetic text, readers construct a basic emotional framework based on their existing knowledge. Textual reading allows readers ample time to reflect on the connotations of the poem. The ancient Chinese philosopher Liu Xie, in his work The Literary Mind and the Carving of Dragons, states, “Emotion is the foundation of literature, while words are the threads of reasoning; when the foundation is correct, the threads follow, and when the reasoning is settled, the words flow” (Liu X. L., 2022). The text itself contains a wealth of emotional elements, and in the process of interpreting the poem, readers also experience a process of emotional sublimation. For instance, in the sentence “孤帆远影碧空尽, 唯见长江天际流” (Green hills on either bank rise opposite and near, A lone sail appears, approaching the sun’s frontier.), the lone sail and faraway blue sky in traditional Chinese culture represent feelings of coldness and solitude, while the use of “唯见” (appears) in the contrast highlights the poet’s melancholic mood, revealing a sense of solitude and loss. Therefore, textual reading and textual analysis occur almost simultaneously, making it easier to evoke ER in readers.

The fourth possibility lies in the richness of the meanings of Chinese characters within the visual system. The same character can express different emotions and imagery depending on the poet’s creation. In textual reading, the words are more likely to evoke associations and thoughts in readers, thus generating a stronger emotional response (Guo, 2018). For example, in the sentence “两岸青山相对出, 孤帆一片日边来” (Green hills on either bank rise opposite and near, A lone sail appears, approaching the sun’s frontier.), words like both banks, green hills, lone sail, and setting sun may be neutral in isolation, but when combined with the imagery, they convey a profound emotional depth.

#### Auditory modality

The first possible explanation is the subjectivity of auditory information. The auditory channel primarily uses sound and rhythm to express emotions (Belfi, 2019), which may have a lesser impact on ER. For example, in the sentence “日照香炉生紫烟, 遥看瀑布挂前川” (Sunlight strikes the Incense Burner Peak, and purple smoke arises, From afar the waterfall seems like a hanging veil across the stream.), the word 香炉 (Burner Peak) might be overlooked during auditory processing of information, and different individuals may have different emotional reactions to the same sound or rhythm. Some might associate the neutral word 香炉 with a certain scene, while others may have no associations at all.

The second possible explanation is the semantic nature of auditory information. Processing semantic information requires more cognitive resources. The brain must transform and understand the content of the poetry, which takes time. In this process, if poetry is presented quickly in an auditory form, it can make it more difficult for individuals to grasp the underlying emotions and form ER (Piţur and Miu, 2025). For example, in the sentence “飞流直下三千尺, 疑是银河落九天” (Its torrent plunges straight down three thousand feet, As if the Milky Way were falling from the ninth heaven.),the scene of 落九天 (straight down three thousand feet) appears unclear in the auditory format, and individuals may find it difficult to form a clear mental image, resulting in a more neutral ER.

#### Visual-auditory modality

In the visual-auditory modality, the integration of visual and audio may improve comprehension of the poem. The visual modality focuses on spatial structure and imagery, while the auditory modality emphasizes temporal changes and sound features. Together, they provide a more comprehensive understanding of the poem (Coutinho and Scherer, 2017). For example, in the sentence “床前明月光, 疑是地上霜” (Before my bed, the moonlight glistens bright, As if the ground were covered with frost’s white.), the visual imagery of the bright moon becomes more vivid, while the passage of time in the auditory channel adds an additional dimension. Together, they contribute to the deep emotional layers of the poem.

### The impact of background information on the emotional resonance of poetry

In line with hypothesis H2, our study found that both background music and background images enhanced the ER of the poetry.

#### Imagery interpretation and cultural empathy

During the process of interpreting poetry, readers encounter keywords that carry the emotional essence of the entire poem. The surrounding imagery serves to enhance this emotional focal point (Li, 2012). For example, in the sentence “举头望明月, 低头思故乡” (I raise my head to gaze upon the moon, Then lower it, recalling my hometown soon.), the emotional core lies in the word “思” (gaze). The imagery of the moon and bowing my head is recreated in the background images, which deepens the interpretation of the imagery and highlight the emotional point. This progression moves from basic reading and understanding of the text to a more holistic perception of the work. It deepens the higher-level understanding of the poem, thereby fostering a deeper comprehension of the poem.

#### Imagery representation, and cultural metaphors

These images are carriers of the poet’s emotions and thoughts, containing rich cultural backgrounds and symbolic meanings (Kim and Wedell, 2016). The poet’s time, environment, and influences shape the creation of these images. The imagery in poetry goes beyond words, and background images serve to re-present these poetic visuals, blending the poet’s inner world with the surrounding environment. This enhances the emotional expression of the poetry, making it easier for readers to ER (Marin and Leder, 2018). For instance, in the sentence “举头望明月, 低头思故乡” (I raise my head to gaze upon the moon, Then lower it, recalling my hometown soon.), the imagery of the bright moon and the action of looking up are clearly depicted. The cultural metaphor of the moon symbolizing longing and reunion is made tangible, which helps readers better understand the cultural meaning of the poem and increases their emotional connection with the text.

#### Emotional deepening, and cultural fusion

Playing ancient Chinese guqin music from the Tang Dynasty while reading the poetry transports individuals back to the era of Li Bai, allowing them to immerse themselves more deeply in the poet’s time (Bennett and Ginsborg, 2018). This is not just an auditory experience but a profound spiritual journey. The guqin music itself represents purity and transcendence, symbolizing the noble and refined qualities of the time. It enhances the emotional depth of the poem by enriching its artistic expression, representing both poetic sentiment and beauty (Menninghaus et al., 2018). During the reading process, the rhythm, melody, and tranquility of the music allow the reader to feel the depth and richness of traditional Chinese culture, making it easier to understand the poetic imagery and atmosphere, thus enhancing their comprehension of the poem’s artistic essence (Juslin et al., 2023).

### The impact of sensory modalities and background information on the emotional resonance of poetry

#### Situated conceptualization

In line with hypothesis H3, according to the situated conceptualization theory, individuals construct psychological representations of time, space, entities, and their relationships while understanding and experiencing poetry (Antović, 2021). In the process of appreciating poetry, sensory modalities provide rich perceptual dimensions that form the preliminary imagery of the poem. Background information, on the other hand, provides insights into the poet’s era and cultural background, offering a broader framework. This allows the poem to be interpreted within a broader cultural background, thereby deepening its emotional meaning. For example, in the sentence “故人西辞黄鹤楼, 烟花三月下扬州” (An old friend takes leave westward from Yellow Crane Tower, In the misty, blossom-filled third month, bound for Yangzhou’s bower.), when readers see or hear the term “黄鹤楼” (Yellow Crane Tower), there is an immediate emotional recognition. As a famous cultural landmark in China, the Yellow Crane Tower transcends mere scenery and carries deep cultural significance, representing a longing for home. When the imagery of the Yellow Crane Tower reappeared in the background image, readers were instantly transported back to the poet’s time. Coupled with the serene sound of guqin music, readers felt fully immersed in Li Bai’s era, completely entering the scene. This fusion of sensory and cultural background enhanced ER, allowing the reader to experience the poem’s emotional depth to its fullest.

#### Multisensory synesthesia

In classical Chinese aesthetics, synesthesia refers to the mutual blending, transformation, and fusion between different sensory experiences, which enhances readers’ ER with imagery (Sheng and Wu, 2024). The creation of artistic conception in poetry often relies on the integration of sound (specifically music in this study), color (specifically background images here), and meaning (the inherent connotations and emotions of the poem itself), achieving the artistic conception of painting in poetry, sound in painting, and emotion in sound (Liu, 2021). For example, in the sentence “天门中断楚江开, 碧水东流至此回” (The Mount Tianmen Pass splits the Chu River wide, Where jade waters eastward flow and then turn aside.), the emotions and imagery carried by “Mount Tianmen” are guided by background information. When the distant and clear sound of guqin music and the imagery of Mount Tianmen in the background image reappear, readers not only see the landscape depicted in the poem but also hear the emotional rhythm behind it. This aesthetic experience not only deepens the understanding of the poem’s emotional meaning but also reflects a return to traditional Chinese aesthetics. Although the term synesthesia often holds explanatory power in cross-cultural aesthetic studies (non-native Chinese speakers) as well (Simner, 2005), we can further explore this in future research.

## Conclusion

In the contrast between visual and auditory modalities, the results indicate that the visual modality, through its direct imagery presentation, significantly dominates the activation of ER, and the ER of poetry depends not only on the performance of sensory modalities but also on the collaborative effect of different background information, highlighting the integrative and layered nature of perceptual experience. This finding deepens our understanding of the core role of cross-modal interaction in ER.

### Limitation

Despite this study’s exploration of different sensory modalities and background information on ER in poetry, several limitations remain. The experimental design includes five conditions, and the sample size is relatively limited, which may restrict the generalizability of the results. Future research can expand the sample size and include representative groups across different age ranges and populations to enhance the external validity of the findings. Additionally, the types of background music and background images used in this study are relatively limited and do not cover a richer variety of background information. Subsequent studies should consider introducing diversified audiovisual background materials to further investigate whether subtle differences among different background information exist. Finally, ER exhibits strong individual variability. Future research needs to explore individual characteristics (such as cultural background and personal experiences) as moderating factors to gain a more comprehensive understanding of this complex psychological process.

## Data Availability

The raw data supporting the conclusions of this article will be made available by the authors, without undue reservation.
